# Effect of nutrients deficiency on biofilm formation and single cell protein production with a purple non-sulphur bacteria enriched culture

**DOI:** 10.1016/j.bioflm.2022.100098

**Published:** 2022-12-15

**Authors:** S. Shaikh, N. Rashid, U. Onwusogh, G. McKay, H.R. Mackey

**Affiliations:** a Division of Sustainable Development, College of Science and Engineering, Hamad Bin Khalifa University, Qatar Foundation, Doha, Qatar; b Qatar Shell Research and Technology Centre, Tech 1, Qatar Science and Technology Park, Doha, Qatar; c Department of Civil and Natural Resources Engineering, University of Canterbury, Private Bag 4800, Christchurch, 8140, New Zealand

**Keywords:** Fuel-synthesis wastewater, Purple non-sulphur bacteria, Single cell protein, Biofilm, Nutrients deficiency, PNSB, Purple non-sulfur bacteria, FSW, Fuel synthesis wastewater, SCP, Single cell protein, TS, Total solids, COD, Chemical oxygen demand, SEM, Scanning electron microscope

## Abstract

Purple non-sulphur bacteria (PNSB) are of interest for biorefinery applications to create biomolecules, but their production cost is expensive due to substrate and biomass separation costs. This research has utilized fuel synthesis wastewater (FSW) as a low-cost carbon-rich substrate to produce single-cell protein (SCP) and examines PNSB biofilm formation using this substrate to achieve a more efficient biomass-liquid separation. In this study, PNSB were grown in Ca, Mg, S, P, and N-deficient media using green shade as biofilm support material. Among these nutrient conditions, only N-deficient and control (nutrient-sufficient) conditions showed biofilm formation. Although total biomass growth of the control was 1.5 times that of the N-deficient condition and highest overall, the total biofilm-biomass in the N-deficient condition was 2.5 times greater than the control, comprising 49% of total biomass produced. Total protein content was similar between these four biomass samples, ranging from 35.0 ± 0.2% to 37.2 ± 0.0%. The highest protein content of 44.7 ± 1.3% occurred in the Mg-deficient condition (suspended biomass only) but suffered from a low growth rate. Overall, nutrient sufficient conditions are optimal for overall protein productivity and dominated by suspended growth, but where fixed growth systems are desired for cost-effective harvesting, N-deficient conditions provide an effective means to maximize biofilm production without sacrificing protein content.

## Highlights

• Effect of various nutrients deficiency on PNSB biofilm formation was investigated.
• Nitrogen-deficiency and nutrient-abundance (control) induced biofilm formation.
• Nitrogen-deficient condition led to low suspended growth but high biofilm growth.
• The maximum protein content was obtained in magnesium-deficient condition.
• The minimum protein content was obtained in calcium-deficient condition.

## Graphical abstract

**Figure:**
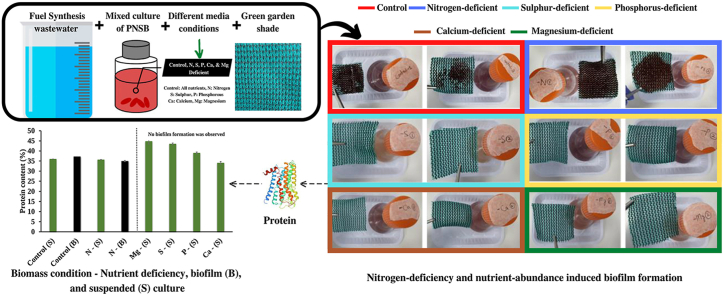

## 1 Introduction

Growing population and consumption patterns, in conjunction with climate change, have led to increasing scarcity of water, energy, and other resources. This has led to the uptake of more energy efficient wastewater treatment processes with a target of resource recovery [1]. Amongst the many innovative technologies addressing the recovery of valuable resources from wastewater, biological approaches offer the strongest promise for efficiently recovering valuable resources from dilute streams. These processes provide a cost-effective and diverse method of concentrating and transforming wastewater resources into valuable products. For instance, biohydrogen can be produced through photo and dark fermentation processes [2], and biogas via anaerobic digestion processes [3] whereas direct conversion of lipidic biomass into biodiesel has also received interest in the past decade [4]. The production of bioplastics from organic acids is another option for carbon recovery [5]. For a holistic recovery of both nutrients and carbon from wastewater single cell protein (SCP) production is a favourable option, which may be utilized as the next generation of fertilizers, animal feed, and feed additives [6].

SCP is protein-rich unicellular biomass or protein isolated from algae, yeast, fungi, or bacteria. It can replace animal and human protein sources [7]. SCP typically contains other essential dietary components such as vitamins, fats, minerals, and carbohydrates [8]. Apart from the nutritional benefits [9], it is considered one of the most promising approaches to improve global food security [10]. Microbial protein can be produced indoors and on non-arable land, unlike plant-based protein [11]. It is predicted that by 2050 microbial protein could replace 10–20% of animal and crop-based protein [12]. SCP production typically entails the following steps: (a) preparing nutritional medium, possibly from agricultural or industrial process residues, (b) cultivation, either through liquid or solid-state fermentation, (c) separating and concentrating and typically drying the SCP, and (d) finally processing SCP into ingredients and products [13].

A major cost of SCP production is the substrate, making integration with the bioremediation of agricultural or industrial waste streams an attractive option. This can be achieved with various bacteria including chemoheterotrophs, microalgae and anoxygenic photosynthetic bacteria [14]. The latter group of bacteria have the advantage of anaerobic growth and easy enrichment using specific wavelengths of light. Within the anoxygenic photosynthetic bacteria, purple non-sulphur bacteria (PNSB) are primary candidates of interest. This is due to their photoheterotrophic metabolism, which allows high substrate conversion yields [15], protein rich biomass which can reach up to 72% crude protein [16], and the ability to produce many value-added products. These include 5-aminolevulinic acid [17], coenzyme Q10 [18], polyhydroxyalkanoates [19], carotenoids, and bacteriochlorophylls [20]. PNSB biomass is a desirable source of SCP for aquaculture as its protein amino acid composition is similar to that of soyabean proteins including essential amino acids for aquatic organisms [21]. Moreover, SCP from PNSB has shown additional health benefits in aquaculture studies, such as increased pathogenic resistance for white shrimp (*Litopenaeus vannamei*) [22]. However, its commercial production is limited because of excessive costs associated with substrate and biomass separation [23].

Commonly used carbon sources such as sugars and volatile fatty acids are not economically affordable. Therefore, low-cost carbon sources such as wastewater from soyabean oil, olive mill, cheese whey, and molasses industries are of interest for SCP production [24]. Gao et al. used soy molasses to produce SCP from *Candida tropicalis* [25]*.* Dubois Frigon used an acid whey to produce SCP using mixed yeast culture. He and colleagues reported that the biomass amino acid profile was suitable with respect to the Food and Agricultural Organization (FAO) protein nutrition guidelines for various farmed livestock species demonstrating the potential of SCP production from waste substrates [26]. However, many other non-agri-food waste streams exist that could potentially be used for SCP production. In this study, FSW is used as a non-agricultural waste source because it has high organic content and low toxicity [27]. FSW is a by-product from the Fischer-Tropsch process combining carbon monoxide and hydrogen over a catalyst into alkanes. The world’s largest gas to liquids plant utilizing Fischer-Tropsch process produces around 140,000 barrels of oil equivalent products each day with a corresponding production of organic-laden by-product water of 45,000 m^3^/day [28]. Current global FSW production is around 270,000 barrels daily [29], placing current global FSW production around 32 million m^3^/year. This value is expected to increase significantly in the coming decades if oil prices increase [29]. Most current FSW treatment is done using aerobic biological oxidation, converting valuable organics to carbon dioxide with relatively high energy inputs to do so. Therefore, its valorisation using alternate treatment approaches such as PNSB is desirable.

In addition to high substrate costs, biomass separation is a critical factor in the economics of the process due to the relatively dilute cultures typical in phototrophic systems [30]. For instance, Alloul et al. estimated the cost of SCP as approximately $11–22/kg dry weight, even though cultivated using low cost brewery wastewater as substrate [31]. SCP harvesting from bacteria is costly because of the smaller cell size, requiring cells to be flocculated to produce a higher solids slurry prior to centrifugation or flotation [32]. Harvesting challenges can be overcome with biofilm-based systems, providing high biomass concentrations and simple harvesting techniques [30]. Biofilms can be harvested at cell densities up to 100 times more concentrated (>100 g/L) than suspended biomass (<2.0 g/L), which reduces dewatering and harvesting costs [33,34]. Bacterial biofilm growth is influenced by several parameters, including pH, temperature, O_2_ levels, hydrodynamics, osmolarity, the presence of particular ions, carbon source, and nutrients [35]. Various studies in the literature reported that the nutrients concentration in the medium can affect the biofilm formation [36,37]. However, the response to nutrient availability differs both from studies at species level and in mixed cultures. For instance, studies with *Pseudomonas putida* [38], *Citrobacter* sp. [39], and mixed cultures [40] found that a higher concentration of nutrients promotes biofilm formation. In contrast, independent studies with *Listeria monocytogenes* [41]*,* and both individual and combined cultures of *Citrobacter freundii* and *Enterobacter cloacae* [42], found higher biofilm formation when at least one nutrient was limited. What is clear from these studies is that nutrient availability is important in defining cell metabolism and the production of extracellular polymers that define cell surface and adhesion properties. However, to the authors knowledge, no studies have reported the effect of nutrient deficiency on biofilm formation by mixed cultures dominated by PNSB.

Therefore, this study investigates nutrient deficiency effect on PNSB biofilm formation and evaluates the relative performance of this biofilm compared to suspended cultures grown on FSW for the production of SCP. To our knowledge, this is the first study investigating the effect of the deficiency in various nutrients on PNSB biofilm formation and single cell protein production. Key questions to be addressed in the study were a) What effect does deficiency of different nutrients have on FSW treatment? b) Which nutrient deficient conditions can promote PNSB biofilm formation? c) What is the effect of different nutrient deficiencies on PNSB growth? and d) Does the deficiency of certain nutrients impact on cellular protein content?

## 2 Materials and methods

### 2.1 Green shade biofilm support

**Fig. 1:**
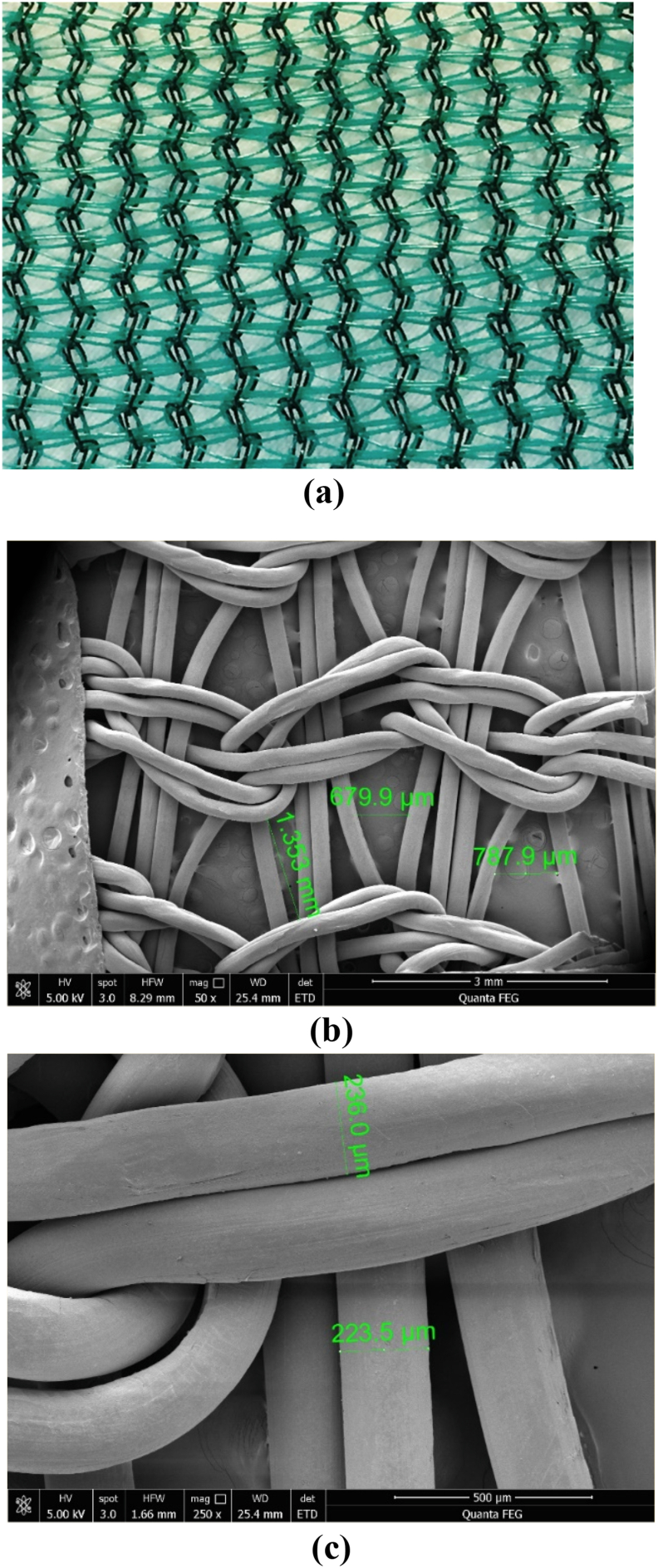
Images of green shade (a) Photo of green garden shade used in this study for biofilm development (b) SEM image of green garden shade at 50× magnification (c) SEM image of green garden shade at 250× magnification.

Green garden shade was used as a biofilm support in this study after some initial trials with a variety of materials. It is porous, providing sites for microbial adhesion and allowing good light penetration to the biofilm from both directions. It is also economical, thin, and readily available in the market (Fig. 1). The green shade morphology was viewed by scanning electron microscopy (SEM) using a FEI Quanta 250 FEG at 5.0 kV acceleration voltage. Prior to imaging, samples were sputter coated with 8 nm of gold.

### 2.2 PNSB substrate and media preparation

A mixed culture of PNSB was used as inoculum, which was initially seeded from a mixture of coastal seawater and a freshwater landscaping pond. This seed was cultivated for over a year in the laboratory on media containing FSW and nutrients according to ATCC 2728 media. The nutrients added in the seeding of PNSB was KH_2_PO_4_ (3.03 g/L), NH_4_Cl (3.03 g/L), NaHCO_3_ (4.29 g/L), ATCC trace minerals supplement (MD-TMS) (1 mL/L), and ATCC vitamin supplement (MD-VS) (1 mL/L). Trace elements solution and vitamin solution compositions are given in Table 2. Similarly, in this experiment FSW was used as a carbon substrate. The FSW was selected as a carbon source because it is clear, with no visible colloidal or settleable matters. The chemical oxygen demand (COD) of the FSW used in this study was 6767 ± 272 mg/L and was diluted five times for use in the experiments without sterilization. The characteristics of the FSW are reported in Table 1. As FSW is the result of the synthetic conversion from syngas to alkanes it contains little to no chemicals other than organics consisting of C, H and O [27]. Other nutrients such as nitrogen, phosphorus, trace elements, and vitamins are also necessary for PNSB growth [43], but must be added, making it an ideal substrate for the study of nutrient deficiency.

**Table 1:** Measured characteristics of the FSW prior to nutrient addition.

Parameter	Concentration	Unit
pH	3.6 ± 0.2	–
Conductivity (μS/cm)	430 ± 1	μS/cm
Total dissolved solids	200 ± 10	mg/L
Total COD	6767 ± 272	mg/L
Total organic carbon	2013 ± 115	mg/L
Total carbon	2620 ± 134	mg/L
Inorganic carbon	606 ± 19	mg/L
Total nitrogen	0	mg/L
Organic acids		
Acetate	1085 ± 2.6	mg/L
Propionate	323 ± 1.7	mg/L
Iso-butyrate	21.5 ± 0.2	mg/L
Butyrate	275 ± 0.5	mg/L
Iso-valerate	28 ± 0.0	mg/L
Valerate	237 ± 2.9	mg/L

**Table 2:** Composition of ATCC trace mineral and vitamin supplement solutions.

ATCC trace mineral supplement solution	
Element	Concentration (g/L)
Ethylenediamine-tetraacetate-di-sodium-salt	0.5
Iron (II) sulphate heptahydrate	0.1
Boric acid	0.010
Cobalt (II) nitrate hexahydrate	0.1
Manganese (II) sulphate hydrate	0.5
Zinc sulphate heptahydrate	0.1
Nickel (II) chloride hexahydrate	0.020
Sodium molybdate dihydrate	0.010
Copper (II) sulphate pentahydrate	0.010
Magnesium sulphate heptahydrate	3.0
Sodium chloride	1.0
Calcium chloride anhydrous	0.1
Aluminium potassium sulphate dodecahydrate	0.010
Sodium tungstate dihydrate	0.010
Sodium selenite	0.001
ATCC vitamin supplement solution	
Element	Concentration (mg/L)
Folic acid	2
Pyridoxine hydrochloride	10
Riboflavin	5
Biotin	2
Thiamine hydrochloride	5
Nicotinic acid	5
Calcium pantothenate	5
Vitamin B12	0.1
p-aminobenzoic acid	5
Thioctic acid	5
Monopotassium phosphate	900

Therefore, nutrients that were added in seeding of PNSB were also added in this experiment. However, in each of the nutrient-deficient conditions, the nutrient of interest was excluded. The control contained all nutrients as mentioned above.

### 2.3 Culturing test procedure

The effect of nutrients deficiency on biofilm growth was investigated by cultivating cultures of PNSB on FSW prepared as described in Section 2.2 which were deficient in one of each of the following elements: nitrogen (N), phosphorous (P), sulphur (S), calcium (Ca) and magnesium (Mg). A control was also considered containing all nutrients. The starting absorbance of all conditions was set as 0.2 ± 0.0 OD_420._The test was conducted under anaerobic conditions in sealed Erlenmeyer flasks (250 mL) with a working volume of 250 mL. The flasks were placed in a shaking incubator (Innova 44, New Brunswick, Canada) at 200 rpm under continuous lighting (7 W/m^2^). A temperature of 35 °C was used for the study since growth of PNSB is optimal at 25–35 °C [44]. This temperature is also similar to daytime temperatures in the study location, that is Qatar, which ranges between 30 and 40 °C for most of the year. The initial pH of all conditions was 7.1 ± 0.0 with no control of pH during the study duration. A piece of green garden shade (12 cm^2^) was suspended in each flask as a supporting material for biofilm development. The experiment was conducted for 32 days, during which both biofilm growth on the green shade as well as suspended growth in the same flask could occur. Each nutrient culture was performed in duplicate.

### 2.4 Determination of PNSB biomass

The PNSB growth in suspended culture was measured by absorbance at 420 nm using a UV-3600 plus spectrophotometer (Shimadzu, Japan) nm. Measurements were taken at day 1, 5, 13, 18, 25, and 32. A wavelength of 420 nm was chosen as this corresponds to absorbance by carotenoids present in the PNSB giving a better indicator of the growth of PNSB [45], compared with all bacteria typically measured at around 600 nm. At the end of the experiment gravimetric analysis of total solids (TS) was conducted for both the suspended and biofilm fractions of the sludge culture using standard methods [10]. Biofilm was first released from the green shade using a spray bottle with a known volume of distilled water.

### 2.5 Analysis of effluent

The solution pH was measured by a pH meter (Thermo Scientific, USA). Samples of the effluent wastewater were centrifuged at 10,000 rpm for 10 min in a centrifuge (Sorvall LYNX 6000, Thermo Scientific, USA) and filtered through 0.45 μm polyethersulfone syringe filters (Nalgene, Fisher Scientific, USA) to obtain the supernatant for the analysis of COD. The COD was measured by USEPA Reactor Digestion Method 8000 using Hach (USA) high range Test’N’tube kits [46]. The pH and COD of samples were analysed at day 1, 5, 13, 18, 25, and 32. The COD removal and COD removal efficiency were calculated by Eq. (i) and Eq. (ii), respectively.

(i) $$COD\;removal\;\left(mg/L\right)={COD}_{day1}-{COD}_{day32}$$

(ii) $$COD\;removal\;efficiency\;\left(\%\right)=\frac{{COD}_{day1}-{COD}_{day32}\;}{{COD}_{day1}}*100$$

### 2.6 Extraction and determination of single cell protein (SCP)

SCP was extracted at the end of the experiment by the alkaline extraction method reported by Perovic et al. with minor modifications [47]. The 15 mg of lyophilized ground sample was mixed with 10 mL of 0.4 M NaOH and agitated on a compact digital rocker (Model 88880020, Thermo Scientific, USA) at 100 rpm for 30 min. After agitation, samples were sonicated in an ultrasonic bath (CPX1800H-E, Branson, USA) at 70 °C for 2 h followed by digestion at 100 °C for 1 h in a COD digital reactor block (DRB 200, Hach, USA). After samples were cooled down, these samples were centrifuged at a speed of 23,366 g and a temperature of 4 °C, and pellets were discarded. To precipitate the proteins, the pH of the supernatant was adjusted to 3 by adding 1 M HCl, drop by drop. The sample was then centrifuged again, followed by pH adjustment to 12 using 1 M NaOH. The sample was centrifuged a final time and the supernatant was analysed for true protein using the Lowry protein assay method [48].

### 2.7 Data analysis

All tests were conducted in biological duplicates. The statistical differences between samples were analysed using the Welch’s analysis of variance (ANOVA), given the uncertainties in equality of variance associated with small sample sizes. Post-hoc testing of a significant difference was undertaken using the conservative Bonferroni test for similar reasons. The level of significance considered was 5%. Analysis was conducted using JASP.

## 3 Results and discussion

### 3.1 FSW treatment

**Fig. 2:**
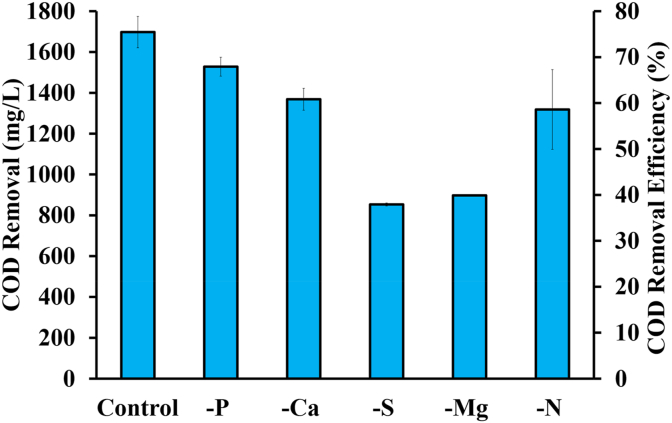
COD removal and efficiency in various nutrients-deficient mediums. The error bars indicate the mean ± standard deviation determined from biological duplicates.

COD removal was the highest in the control condition (1562 ± 71 mg/L), followed by the P-deficient condition (1406 ± 42 mg/L). The COD removal of the Mg-deficient (826 ± 0.0 mg/L) and S-deficient (785 ± 7 mg/L) conditions were considerably lower than in other conditions and almost half that of the control. These removals corresponded to removal rates ranging from 38 ± 0.3% in the S-deficient condition to 75 ± 3.4% in the control (Fig. 2). The COD removal rates in Ca- and Mg-deficient conditions were similar (p > 0.05) but differed from all other conditions (p < 0.05). Magnesium is essential for bacteriochlorophyll synthesis [49] and, therefore, general photoheterotrophic metabolism, explaining its major effect on organic transformation. It is also important for the synthesis of PNSB protoplasm and the cell division mechanism [50]. Similarly, sulphur availability is essential for nitrogenase activity and provides a switch between various carbon utilization or storage mechanisms in the cell [43], but more importantly it is a key element of the ethylmalonyl-CoA pathway, which is involved in carbon fixation, particularly for *Rhodobacter* [51]. The switching of metabolic pathways caused by sulphur deprivation of nitrogenase requires energy to generate the necessary enzymes and may slow down the rate of COD consumption and growth [52]. Although P is a major and critical nutrient for biomass synthesis, the relatively unaffected COD removal under P-deficient conditions may be due to the potential of certain PNSB organisms to store polyphosphate [53,54]. It is possible that the organisms contained stored polyphosphate from the seeding culture, which was cultivated with the presence of phosphate in the medium. The impacts of nitrogen were not as severe as some other nutrients that are typically required at much lesser quantities (on a stoichiometric ratio to carbon consumed), and is most likely due to the nitrogen fixing capability of PNSB [55].

### 3.2 Effect of nutrients deficiency on PNSB growth (biofilm and suspended culture)

**Fig. 3:**
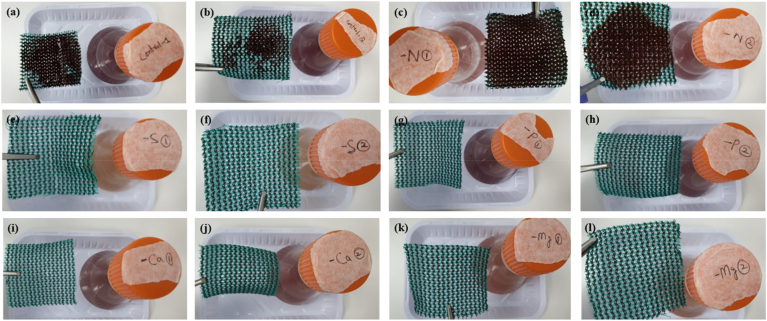
PNSB biofilm formation on green shade at the end of experiment in (a–b) the control, and in (c–d) nitrogen, (e–f) sulphur, (g–h) phosphorous, (i–j) calcium and (k–l) magnesium-deficient conditions.

Biofilm formation was only observed in the N-deficient condition and the control, where the former was notably more prevalent. In both cases, biofilm development occurred in the later portion of the study, visually observed from day 7 onwards. No biofilm formation was observed in the other nutrient-deficient conditions (Fig. 3). Images of the biofilm formation in all conditions from day 7 onwards is provided in the Supplementary Material.

**Fig. 4:**
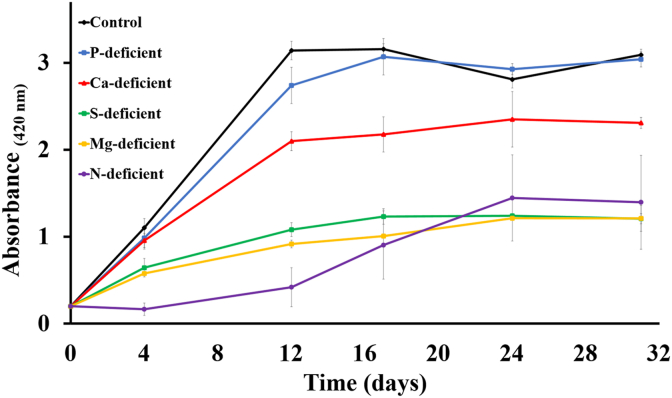
PNSB suspended growth (optical density at 420 nm) in all the different conditions. The error bars indicate the mean ± standard deviation determined from biological duplicates.

Suspended culture growth followed the same trend as the COD removal with the maximum optical density at the final day obtained for the control condition (3.0 ± 0.0 OD_420_) followed by P (3.0 ± 0.0 OD_420_), Ca (2.3 ± 0.0 OD_420_), N (1.4 ± 0.5 OD_420_), Mg (1.2 ± 0.1 OD_420_) and S-deficient (1.2 ± 0.0 OD_420_) conditions (Fig. 4). Sulphate assimilation is critical for synthesis of sulphur containing amino acids such as cysteine, methionine and homocysteine, and may be a reason for the limited growth [56]. Given the importance of sulphur-containing amino acids in aquaculture SCP feeds, sulphur availability must be maintained for both biomass productivity and biomass quality [56]. For N-deficiency, the growth of PNSB was initially the slowest, indicating that the N-deficient condition has a higher lag phase than all other conditions. However, after the lag, which was most likely associated with upregulation of nitrogenase, the culture showed growth comparable to most other conditions.

**Fig. 5:**
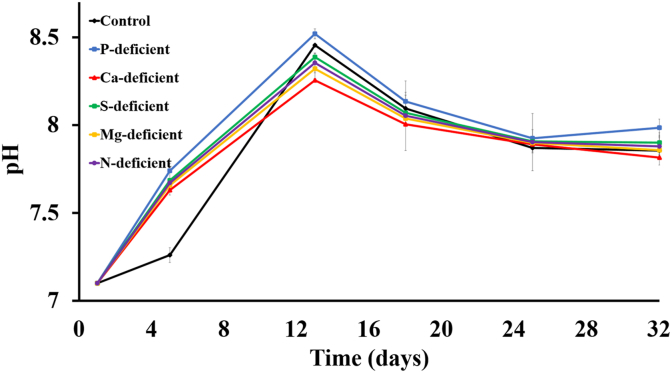
pH profile through the test for each test condition. The error bars indicate the mean ± standard deviation determined from biological duplicates.

The pH, while uncontrolled, was similar between culture conditions (Fig. 5) and therefore not a significant factor in the variation in growth. Biofilm growth occurred later than suspended growth, and therefore seems less influential on the pH. The pH of all different conditions increased with time and reached between 8.2 ± 0.0 (Ca-deficient) and 8.5 ± 0.0 (P-deficient) at day 13. This corresponded with the end of the logarithmic growth phase of the suspended culture in all conditions except N-deficient (Fig. 4), which had a lag phase (Fig. 3). Following this point in the experiment, the pH decreased for all conditions, eventually plateauing just below 8, despite the differences in growth and COD removal achieved in the different samples. The variation in the pH range of the study did not seem to impact the growth significantly.

**Fig. 6:**
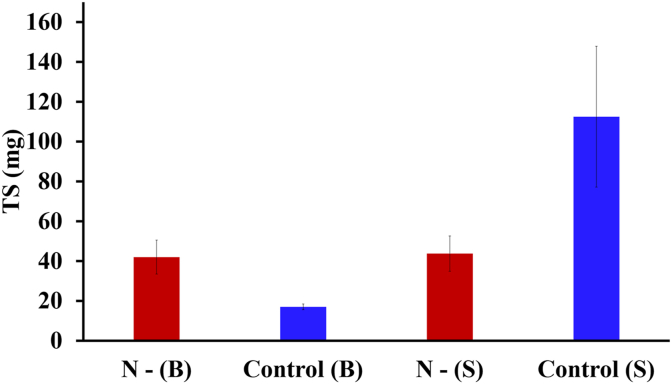
TS of biofilm (B) and suspended culture (S) in nitrogen-deficient (N-) and control condition. The error bars indicate the mean ± standard deviation determined from biological duplicates.

TS results showed that the control condition had high suspended growth (113 ± 35.3 mg) relative to its biofilm growth (17 ± 1.4 mg), with suspended growth accounting for 87% of total biomass (Fig. 6). Comparatively, N-deficient conditions led to low suspended growth (44 ± 8.8 mg) which was similar in magnitude to the biofilm growth (42 ± 8.5 mg). When comparing the biofilm growth between the control and N-deficient conditions, however, the latter was 2.5 times more than that observed in the control, indicating N-deficient conditions promote biofilm growth. However, it should also be noted that total biomass production in the control was 1.5 times more than that achieved in the N-deficient conditions, which is a critical consideration for overall SCP productivity.

### 3.3 Effect of nutrients deficiency on single cell protein production

The protein content in the suspended culture of different conditions varied from 35 to 44%. The maximum protein content was obtained in the Mg-deficient condition followed closely by the S-deficient condition. Mg and S-deficient conditions had the lowest organic conversion and least growth at the end of the experiment (Fig. 4). This means there was a higher amount of nitrogen available in solution for the quantity of biomass produced and may therefore lead to a higher protein content. The minimum protein content was obtained in the Ca-deficient condition. While the exact reasons why Ca is so critical to protein content is unclear, Ca is involved in various cell signalling processes and has been linked to cell division, which is accompanied with intracellular increases in protein, while proteins are also involved in regulating Ca concentrations within a narrow range in the cell [57]. The protein content of Mg- and S-deficient conditions were statistically different (p < 0.05) from all other conditions.

**Fig. 7:**
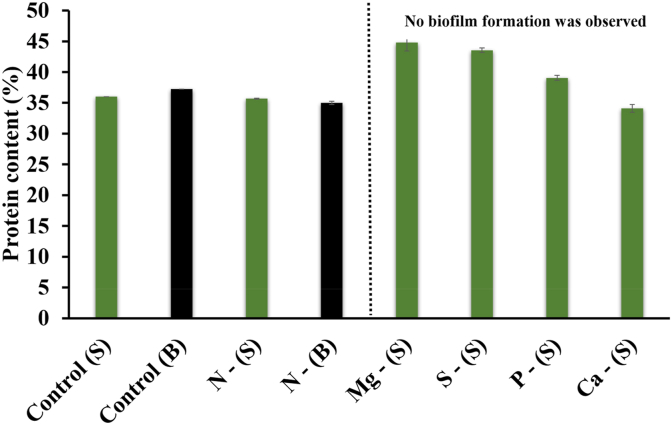
Protein content in biofilm (B) and suspended (S) culture samples of different nutrient-deficient conditions. The error bars indicate the mean ± standard deviation determined from biological duplicates.

The protein content in the biofilm culture varied from 35 to 37% for the control and N-deficient conditions (no other conditions produced biofilm). This was comparable to the values in the suspended biomass, which were 36.0 ± 0.0% for the control and 35.6 ± 0.0% for the N-deficient conditions. No significant difference (p > 0.05) was observed between the four samples (Fig. 7). An interesting point is the similarity in the protein content between the N-deficient and sufficient conditions since nitrogen is an essential component of amino acids [58].

**Fig. 8:**
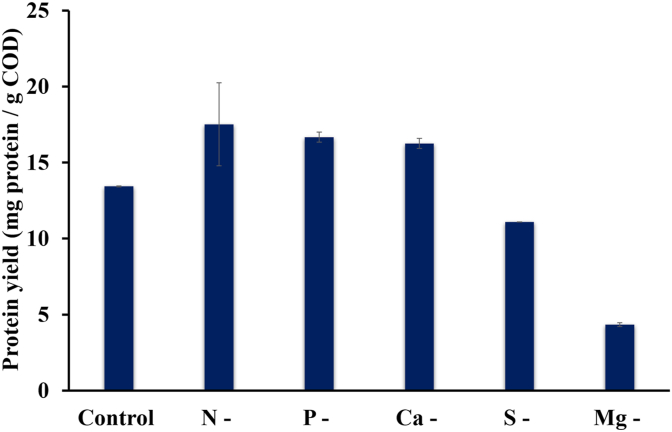
SCP yield in samples of different nutrient-deficient conditions. The error bars indicate the mean ± standard deviation determined from biological duplicates.

Somewhat surprisingly, the highest protein yield was observed in the N-deficient condition (17.5 ± 2.7 mg-protein/g COD consumed) but did not differ significantly (p > 0.05) from P-deficient, and Ca-deficient conditions. Despite having the highest protein content, the Mg-deficient condition had the lowest protein yield (4.3 ± 0.1 mg-protein/g COD consumed). The S-deficient condition, which also had a relatively high protein content had the next lowest protein yield of 11.09 ± 0.0 mg-protein/g COD consumed, due to the low biomass productivity. The protein yield of the Mg-deficient condition differed (p < 0.05) from that of all other tested conditions, and the S-deficient condition also was statistically different from N- and P-deficient conditions (Fig. 8).

The protein contents measured in this study are comparable to those reported in other studies. For instance, Alexandre et al. found 45% protein in *Rhodobacter capsulatus* grown in a synthetic medium [59]. Chumpol et al. investigated the potential of three strains of *Rhodobacter sphaeroides* and *Afifella marina* STW18 (isolated from shrimp ponds) for SCP application. The crude protein of all strains using the Kjeldahl method was 46–54% [22]. Crude protein analysed through the Kjeldahl or Dumas methods are typically higher than the true protein contents measured through the Lowry method used in this study [60]. The protein contents were also comparable to common forms of SCP such as the yeast *S. cerevisiae*, which Tropea et al. found crude and true protein contents of 40% and 38% when grown on food waste [61].

In the last decade, literature reported SCP production from different wastewater resources that required prior treatment. For instance, Saejung & Thammaratana found 60.1% protein from *Rhodopseudomonas* sp. (CSK01 Strain) when cultured in municipal wastewater [62] and Elisa reported crude protein of 67.6% from poultry slaughterhouse wastewater utilizing *Rhodocyclus gelatinosus* [63]. This is the first report of SCP production from fuel-synthesis wastewater. While the protein contents are lower (35–44%) the substrate does not require pre-treatment as does most of the wastewaters. The main benefit of this study, however, is that the natural nitrogen deficiency of FSW lends itself to biofilm-based systems for harvesting, with no impact on cellular protein content of the harvested biomass. This allows not-only an effective means of SCP production without the need for nitrogen inputs, but a means of FSW treatment that does not require addition of this major nutrient. This is important for sustainable protein production given the high energy inputs required to generate urea and ammonia via the Haber-Bosch process that is a feedstock for most protein sources. However, these benefits are likely to be largely outweighed by illumination energy requirements if naturally illuminated systems are not employed [64]. Another important consideration is the biomass production rate. Further study is required to evaluate whether lag times persist under N-deficient conditions, or whether cultures become accustomed which would improve overall productivity, though it is also clear that for total biomass production nitrogen inputs are beneficial. However, these results do demonstrate nitrogen availability should not rule out consideration of a feedstock. Moreover, the effect on amino acid composition should be explored in future to ensure an appropriate alignment with the dietary needs of the intended consuming organisms, noting that differences could be beneficial or detrimental. Lastly, it is expected that the N-deficient condition may lead to improved SCP consistency in a mixed culture environment, since it is an additional selection pressure to limit species diversity.

## 4 Conclusions

Nutrient-deficiency is often considered a suitable way to drive biofilm formation. In this study, N-deficiency was demonstrated as the only nutrient limitation capable of inducing biofilm formation in an enriched PNSB culture treating FSW. All other nutrient-deficient conditions led to no observable biofilm formation, though moderate biofilm development occurred when all nutrients were present together. However, growth under N-limitation showed a notable lag in its suspended growth exponential phase compared to all other tested conditions. Despite this, by the end of the test it had reached moderate biomass concentrations. Similar protein content was observed in the biomass from both N-deficient and control conditions, highlighting that nitrogen addition is not necessary to ensure sufficient protein content in the biomass for SCP application. Moreover, biofilm and suspended growth also showed similar protein content, meaning the selection of biofilm growth to aid separation does not negatively impact the final product, but will require further exploration of suitable reactor designs. The reactor design plays an important role in the sustainability and economic analysis of the process as certain types of reactors may give sustainable outcomes with N-deficiency, while others may require much higher pumping energy, which may (or may not) offset energy and emissions from N fertilizer production. The highest protein contents, occurring with Mg-deficient and S-deficient conditions, was at the cost of overall biomass growth. Further detailed economic analysis, including productivity, market values, harvesting and nutrient supplement costs, are required to understand whether the most desirable SCP route is high overall growth (control), biofilm dominant (N-deficient) or high protein content (Mg-deficient). Therefore, it is concluded that there are many factors that should be explored further to know whether an N-deficient system with biofilm formation may be an effective route for SCP production from nutrient deficient substrate streams or not.

## Funding

This work is supported by the Qatar National Research Fund and the Ministry for Municipality and Environment through the joint Food Security funding program, grant MME01-0910-190029. Open Access funding provided by Qatar National Library (QNL), Qatar.

## CRediT authorship contribution statement

**S. Shaikh:** Conceptualization, Methodology, Formal analysis, Visualization, Writing – original draft. **N. Rashid:** Writing – review & editing. **U. Onwusogh:** Writing – review & editing, Funding acquisition, Resources, Project administration. **G. McKay:** Writing – review & editing, Supervision, Project administration. **H.R. Mackey:** Conceptualization, Methodology, Resources, Supervision, Writing – review & editing, Funding acquisition.

## Declaration of competing interest

The authors declare that they have no known competing financial interests or personal relationships that could have appeared to influence the work reported in this paper.

## Data Availability

Data will be made available on request.

## References

[1] Duarte E., Fragoso R., Smozinski N., Tavares J. (2021). Enhancing bioenergy recovery from agro-food biowastes as a strategy to promote circular bioeconomy. J Sustain Dev Energy, Water Environ Syst.

[2] Akroum-Amrouche D., Abdi N., Lounici H., Mameri N. (2013). Proc 2013 International Renewable Sustainable Energy Conference.

[3] Bhatt A.H., Tao L. (2020). Economic perspectives of biogas production via anaerobic digestion. Bioengineering.

[4] Jafari A., Esmaeilzadeh F., Mowla D., Sadatshojaei E., Heidari S., Wood D.A. (2021). New insights to direct conversion of wet microalgae impregnated with ethanol to biodiesel exploiting extraction with supercritical carbon dioxide. Fuel.

[5] Matassa S., Papirio S., Pikaar I., Hülsen T., Leijenhorst E., Esposito G. (2020). Upcycling of biowaste carbon and nutrients in line with consumer confidence: the “full gas” route to single cell protein. Green Chem.

[6] Abrha H., Cabrera J., Dai Y., Irfan M., Toma A., Jiao S. (2022). Bio-based plastics production, impact and end of life: a literature review and content analysis. Sustainability.

[7] Ritala A., Häkkinen S.T., Toivari M., Wiebe M.G. (2017). Single cell protein-state-of-the-art, industrial landscape and patents 2001-2016. Front Microbiol.

[8] Jacob-Lopes E., Zepka L.Q., Queiroz M.I., Netto F.M. (2006). Protein characterisation of the Aphanothece microscopica Nägeli cyanobacterium cultivated in parboiled rice effluent. Cienc Tecnol Aliment.

[9] Adedayo M.R., Ajiboye E.A., Akintunde J.K., Odaibo A. (2011). Single cell proteins: as nutritional enhancer. Adv Appl Sci Res.

[10] Swetha G., Karunakar R.K., Aruna K., Ramchander M. (2017). Current status on single cell protein (SCP) production from photosynthetic purple non sulphur bacteria. J Chem Pharmaceut Sci.

[11] Henchion M., Hayes M., Mullen A.M., Fenelon M., Tiwari B. (2017). Future protein supply and demand: strategies and factors influencing a sustainable equilibrium. Foods.

[12] Vethathirri R.S., Santillan E., Wuertz S. (2021). Microbial community-based protein production from wastewater for animal feed applications. Bioresour Technol.

[13] Sharif M., Zafar M.H., Aqib A.I., Saeed M., Farag M.R., Alagawany M. (2021). Single cell protein: sources, mechanism of production, nutritional value and its uses in aquaculture nutrition. Aquaculture.

[14] Puyol D., Batstone D.J., Hülsen T., Astals S., Peces M., Krömer J.O. (2017). Resource recovery from wastewater by biological technologies: opportunities, challenges, and prospects. Front Microbiol.

[15] Capson-Tojo G., Batstone D.J., Grassino M., Vlaeminck S.E., Puyol D., Verstraete W. (2020). Purple phototrophic bacteria for resource recovery: challenges and opportunities. Biotechnol Adv.

[16] Kim J.K., Lee B.K. (2000). Mass production of Rhodopseudomonas palustris as diet for aquaculture. Aquacult Eng.

[17] Nunkaew T., Kantachote D., Chaiprapat S., Nitoda T., Kanzaki H. (2018). Use of wood vinegar to enhance 5-aminolevulinic acid production by selected Rhodopseudomonas palustris in rubber sheet wastewater for agricultural use. Saudi J Biol Sci.

[18] He S., Lu H., Zhang G., Ren Z. (2021). Production of coenzyme Q10 by purple non-sulfur bacteria: current development and future prospect. J Clean Prod.

[19] Montiel-Corona V., Buitrón G. (2021). Polyhydroxyalkanoates from organic waste streams using purple non-sulfur bacteria. Bioresour Technol.

[20] Wang H., Yang A., Zhang G., Ma B., Meng F., Peng M. (2017). Enhancement of carotenoid and bacteriochlorophyll by high salinity stress in photosynthetic bacteria. Int Biodeterior Biodegrad.

[21] Garimella S., Kudle K.R., Kasoju A., Merugu R. (2017). Current status on single cell protein (SCP) production from photosynthetic purple non sulphur bacteria. J Chem Pharmaceut Sci.

[22] Chumpol S., Kantachote D., Nitoda T., Kanzaki H. (2018). Administration of purple nonsulfur bacteria as single cell protein by mixing with shrimp feed to enhance growth, immune response and survival in white shrimp (Litopenaeus vannamei) cultivation. Aquaculture.

[23] Anupama Ravindra P. (2000). Value-added food: single cell protein. Biotechnol Adv.

[24] George D.M., Vincent A.S., Mackey H.R. (2020). An overview of anoxygenic phototrophic bacteria and their applications in environmental biotechnology for sustainable Resource recovery. Biotechnol Reports.

[25] Gao Y., Li D., Liu Y. (2012). Production of single cell protein from soy molasses using Candida tropicalis. Ann Microbiol.

[26] Dubois Frigon M. (2020). Acid whey treatment and conversion to single cell protein via aerobic yeast activated sludge. Water Pract Technol.

[27] Boogaard P.J., Carrillo J.C., Roberts L.G., Whale G.F. (2017). Toxicological and ecotoxicological properties of gas-to-liquid (GTL) products. 1. Mammalian toxicology. Crit Rev Toxicol.

[28] Froment T. (2016). http://www.veolia.com/en/our-customers/achievements/industries/oil-gas/qatar-shell-pearl-gtl.

[29] US Energy Information Administration (2017). Global gas-to-liquids growth is dominated by two projects in South Africa and Uzbekistan. Today Energy.

[30] Asri M., Elabed S., Koraichi S.I., El Ghachtouli N. (2019).

[31] Alloul A., Wuyts S., Lebeer S., Vlaeminck S.E. (2019). Volatile fatty acids impacting phototrophic growth kinetics of purple bacteria: paving the way for protein production on fermented wastewater. Water Res.

[32] Anupama Ravindra P. (2000). Value-added food:: single cell protein. Biotechnol Adv.

[33] Ozkan A., Kinney K., Katz L., Berberoglu H. (2012). Reduction of water and energy requirement of algae cultivation using an algae biofilm photobioreactor. Bioresour Technol.

[34] Pierobon S.C., Riordon J., Nguyen B., Ooms M.D., Sinton D. (2017). Periodic harvesting of microalgae from calcium alginate hydrogels for sustained high-density production. Biotechnol Bioeng.

[35] Goller C.C., Romeo T. (2008). Environmental influences on biofilm development. Curr Top Microbiol Immunol.

[36] Cherifi T., Jacques M., Quessy S., Fravalo P. (2017). Impact of nutrient restriction on the structure of Listeria monocytogenes biofilm grown in a microfluidic system. Front Microbiol.

[37] Moreno Osorio J.H., Pollio A., Frunzo L., Lens P.N.L., Esposito G. (2021). A review of microalgal biofilm technologies: definition, applications, settings and analysis. Front Chem Eng.

[38] Rochex A., Lebeault J.M. (2007). Effects of nutrients on biofilm formation and detachment of a Pseudomonas putida strain isolated from a paper machine. Water Res.

[39] Allan V.J.M., Callow M.E., Macaskie L.E., Paterson-Beedle M. (2002). Effect of nutrient limitation on biofilm formation and phosphatase activity of a Citrobacter sp. Microbiology.

[40] Fang W., Hu J.Y., Ong S.L. (2009). Influence of phosphorus on biofilm formation in model drinking water distribution systems. J Appl Microbiol.

[41] Wang Y., Sun L., Hu L., Wang Z., Wang X., Dong Q. (2022). Adhesion and kinetics of biofilm formation and related gene expression of Listeria monocytogenes in response to nutritional stress. Food Res Int.

[42] Thompson L.J., Gray V., Lindsay D., Von Holy A. (2006). Carbon:nitrogen:phosphorus ratios influence biofilm formation by Enterobacter cloacae and Citrobacter freundii. J Appl Microbiol.

[43] Sali S., Mackey H.R. (2021). The application of purple non-sulfur bacteria for microbial mixed culture polyhydroxyalkanoates production. Rev Environ Sci Biotechnol.

[44] Butow B., Dan TB Ben (1991). Effects of growth conditions of acetate utilization by Rhodopseudomonas palustris isolated from a freshwater lake. Microb Ecol.

[45] Chandaravithoon P., Ritchie R.J., Runcie J.W. (2020). Measuring photosynthesis of both oxygenic and anoxygenic photosynthetic organisms using pulse amplitude modulation (PAM) fluorometry in wastewater ponds. J Appl Phycol.

[46] HACH (2019).

[47] Perović M.N., Knežević Jugović Z.D., Antov M.G. (2020). Improved recovery of protein from soy grit by enzyme-assisted alkaline extraction. J Food Eng.

[48] Safi C., Ursu A.V., Laroche C., Zebib B., Merah O., Pontalier P.Y. (2014). Aqueous extraction of proteins from microalgae: effect of different cell disruption methods. Algal Res.

[49] Adessi A., Venturi M., Candeliere F., Galli V., Granchi L., De Philippis R. (2018). Bread wastes to energy: sequential lactic and photo-fermentation for hydrogen production. Int J Hydrogen Energy.

[50] Webb M. (1948). The influence of magnesium on cell division. J Gen Microbiol.

[51] Tang K.H., Tang Y.J., Blankenship R.E. (2011). Carbon metabolic pathways in phototrophic bacteria and their broader evolutionary implications. Front Microbiol.

[52] Hansen T.A., van Gemerden H. (1972). Sulfide utilization by purple nonsulfur bacteria. Arch Mikrobiol.

[53] Fradinho J., Allegue L.D., Ventura M., Melero J.A., Reis M.A.M., Puyol D. (2021). Up-scale challenges on biopolymer production from waste streams by Purple Phototrophic Bacteria mixed cultures: a critical review. Bioresour Technol.

[54] Chen Y.T., Wu S.C., Lee C.M. (2012). Relationship between cell growth, hydrogen production and poly-β-hydroxybutyrate (PHB) accumulation by Rhodopseudomonas palustris WP3-5. Int J Hydrogen Energy.

[55] Maeda I. (2022). Potential of phototrophic purple nonsulfur bacteria to fix nitrogen in rice fields. Microorganisms.

[56] Imhoff J.F., Then J., Hashwa F., Trüper H.G. (1981). Sulfate assimilation in Rhodopseudomonas globiformis. Arch Microbiol.

[57] Dominguez D.C. (2004). Calcium signalling in bacteria. Mol Microbiol.

[58] Reihani S.F.S., Khosravi-Darani K. (2019). Influencing factors on single-cell protein production by submerged fermentation: a review. Electron J Biotechnol.

[59] Poulain A.J., Newman D.K. (2009). Rhodobacter capsulatus catalyzes light-dependent Fe(II) oxidation under anaerobic conditions as a potential detoxification mechanism. Appl Environ Microbiol.

[60] Boulos S., Tännler A., Nyström L. (2020). Nitrogen-to-Protein conversion factors for edible insects on the Swiss market: T. molitor, A. domesticus, and L. migratoria. Front Nutr.

[61] Tropea A., Ferracane A., Albergamo A., Potortì A.G., Turco V Lo, Di Bella G. (2022). Single cell protein production through multi food-waste substrate fermentation. Fermentation.

[62] Saejung C., Thammaratana T. (2016). Biomass recovery during municipal wastewater treatment using photosynthetic bacteria and prospect of production of single cell protein for feedstuff. Environ Technol.

[63] Ponsano E.H.G., Lacava P.M., Pinto M.F. (2003). Chemical composition of Rhodocyclus gelatinosus biomass produced in poultry slaughterhouse wastewater. Braz Arch Biol Technol.

[64] LaTurner Z.W., Bennett G.N., San K.Y., Stadler L.B. (2020). Single cell protein production from food waste using purple non-sulfur bacteria shows economically viable protein products have higher environmental impacts. J Clean Prod.

