# Supplementary material for: Effect of nutrients deficiency on biofilm formation and single cell protein production with a purple non-sulphur bacteria enriched culture
Source: Biofilm. 2022 Dec 15;5:100098. doi: 10.1016/j.bioflm.2022.100098 (PMC9794892; doi:10.1016/j.bioflm.2022.100098)
Supplement: Multimedia component 1 [file mmc1.pdf]

Effect of nutrients deficiency on biofilm formation and single cell protein production with a purple non-sulphur bacteria enriched culture.

Supplementary data

Day 7	Sample 1	Sample 2
Control	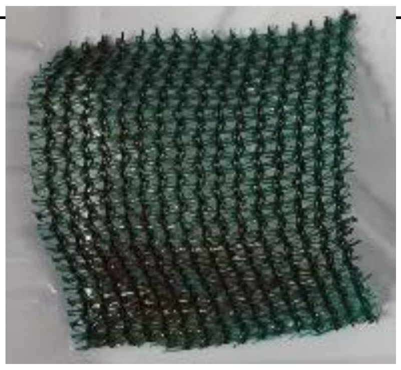	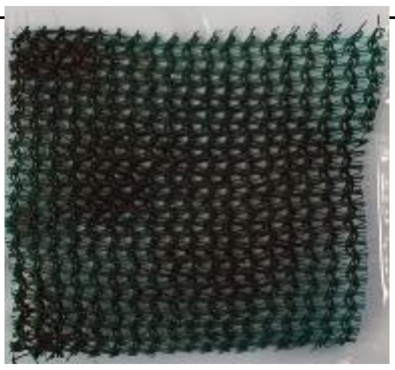
Nitrogen-deficient	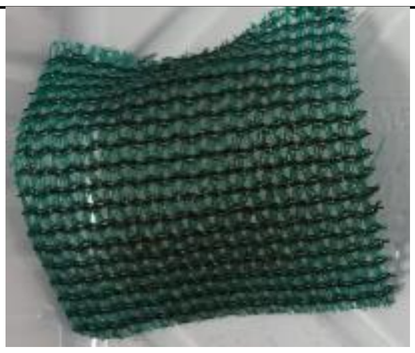	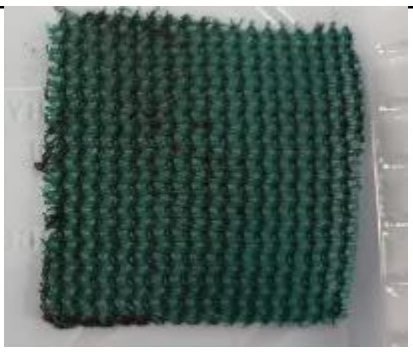
Calcium-deficient	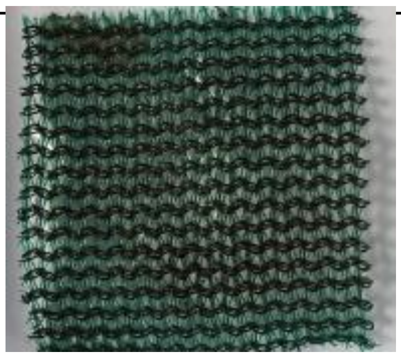	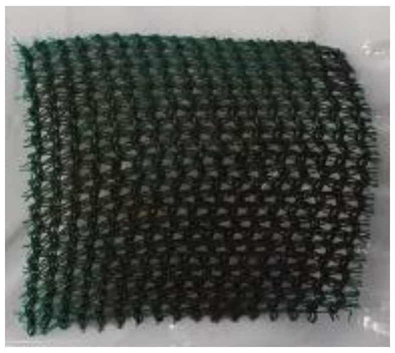
Magnesium-deficient	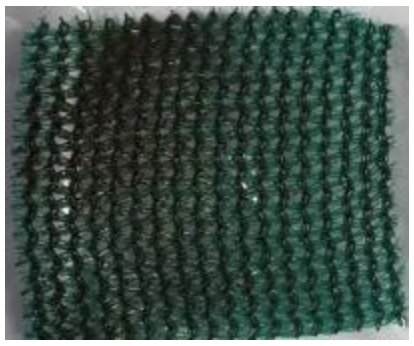	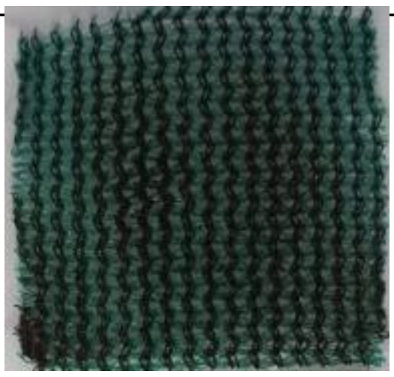
Sulphur-deficient	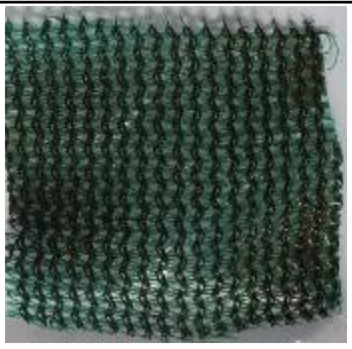	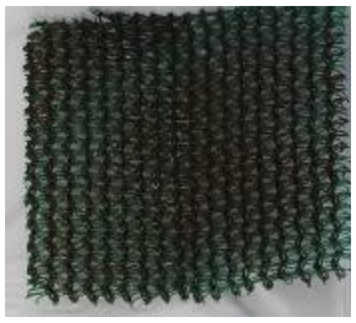
Phosphorous-deficient	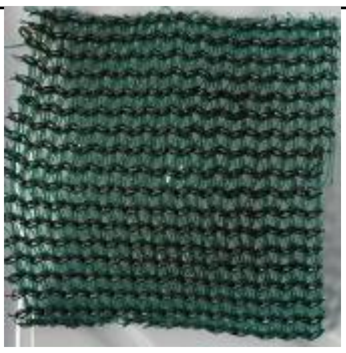	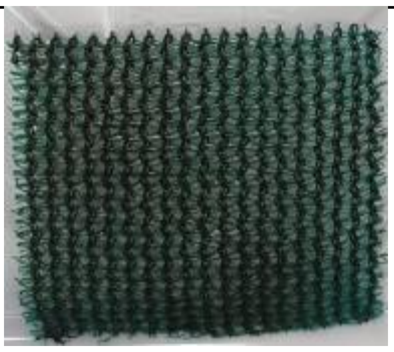

Day 13	Sample 1	Sample 2
Control	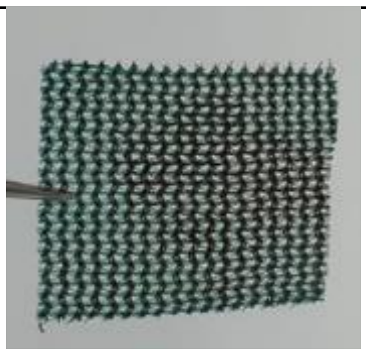	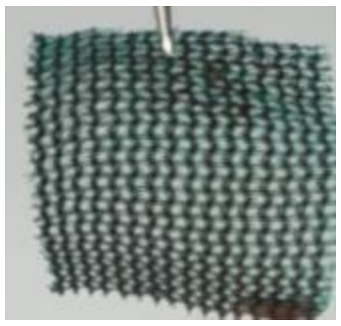
Nitrogen-deficient	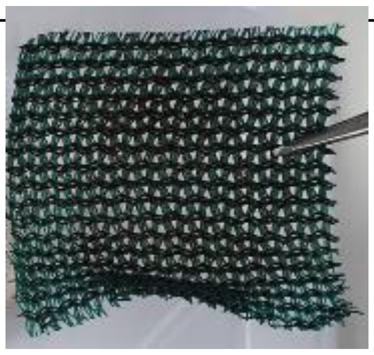	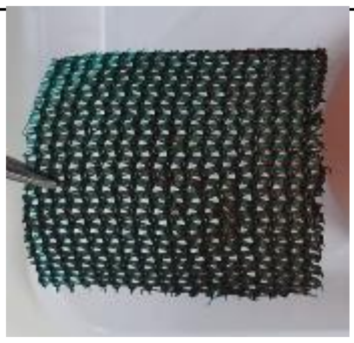
Calcium-deficient	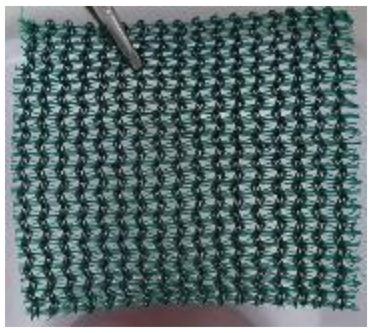	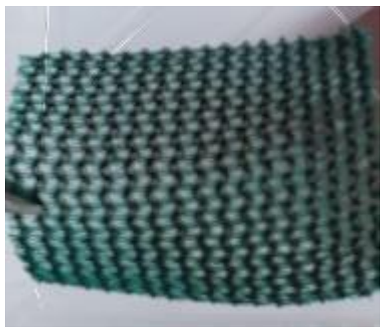
Magnesium-deficient	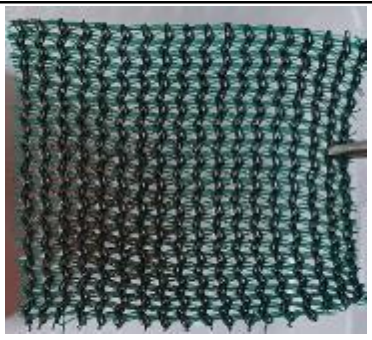	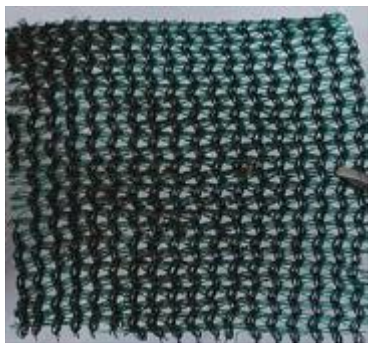
Sulphur-deficient	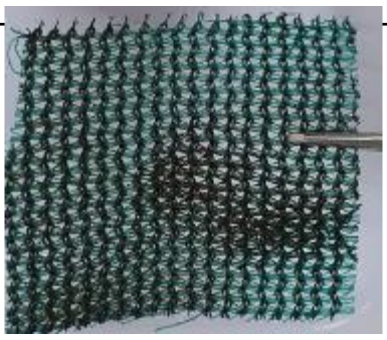	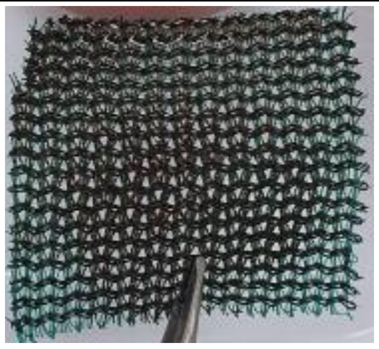
Phosphorous-deficient	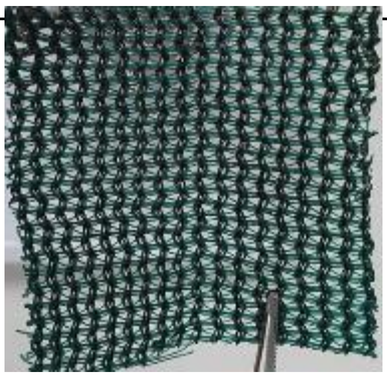	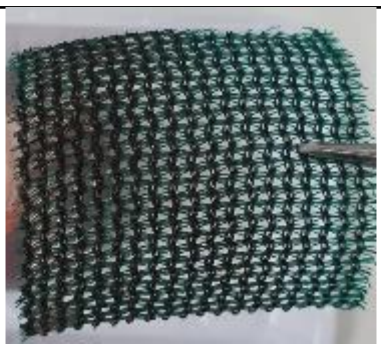

Day 18	Sample 1	Sample 2
Control	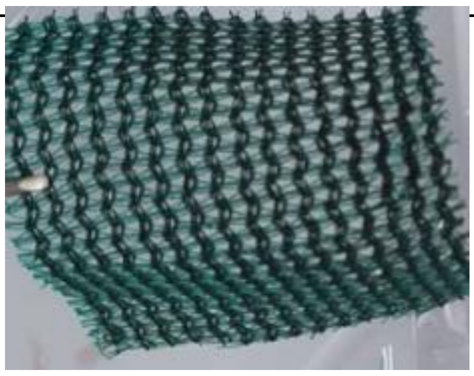	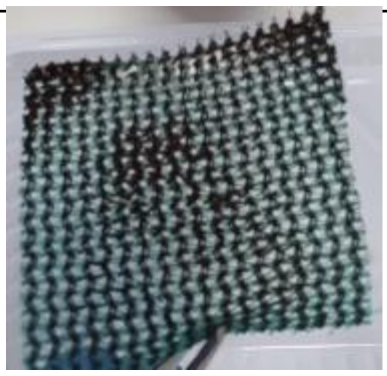
Nitrogen-deficient	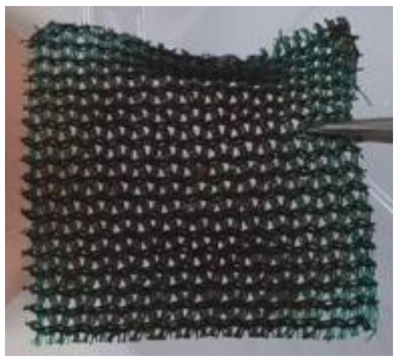	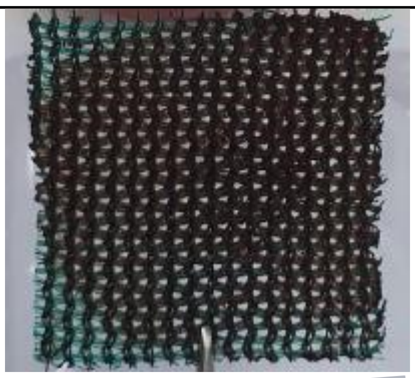
Calcium-deficient	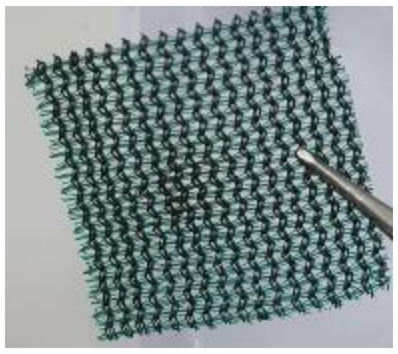	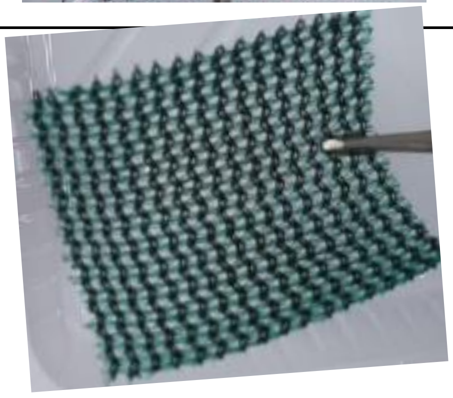
Magnesium-deficient	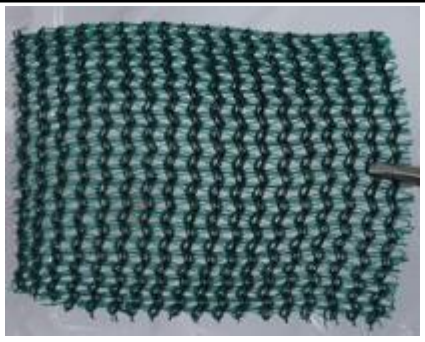	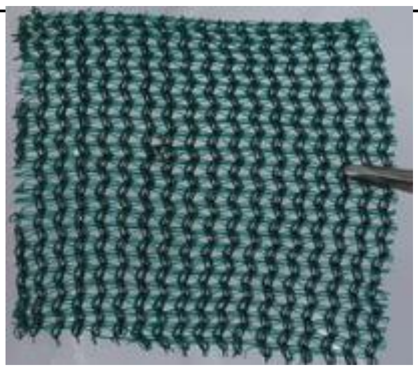
Sulphur-deficient	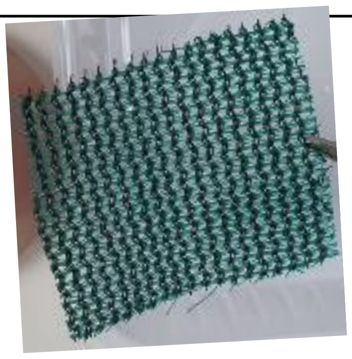	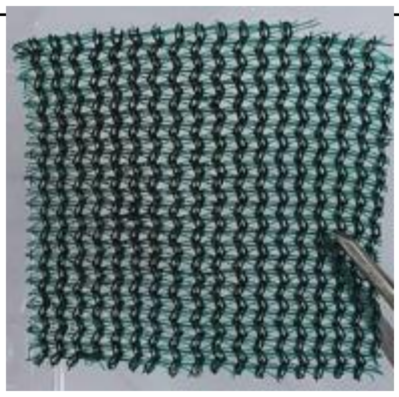
Phosphorous-deficient	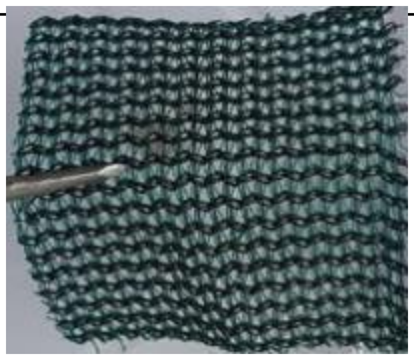	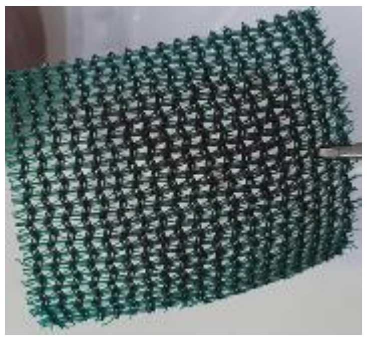

Day 25	Sample 1	Sample 2
Control	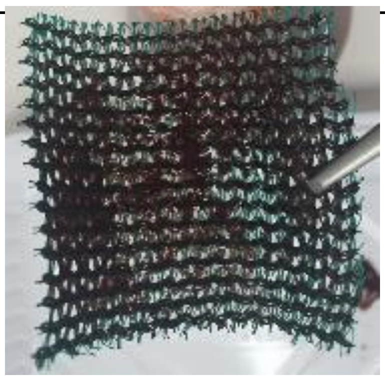	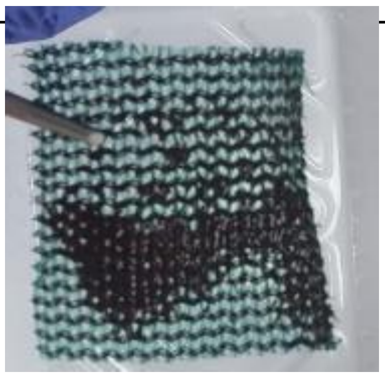
Nitrogen-deficient	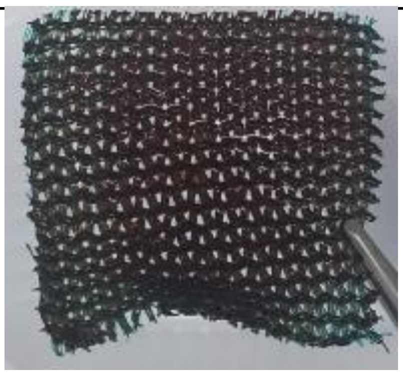	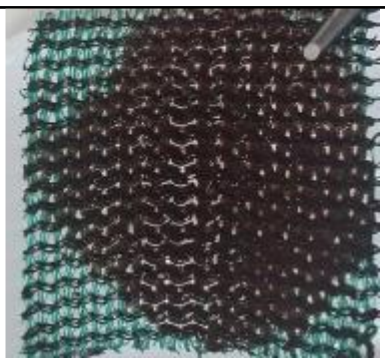
Calcium-deficient	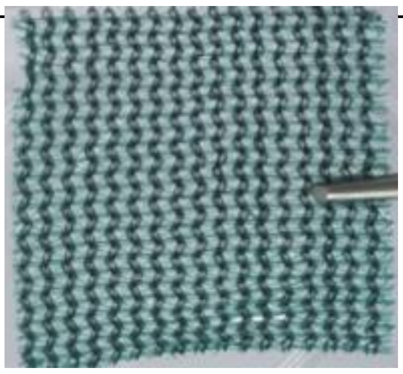	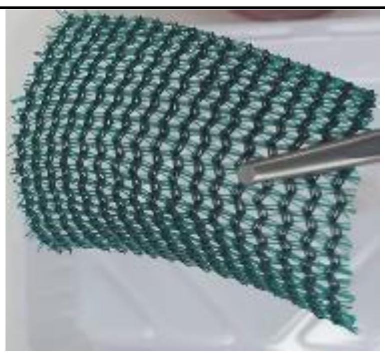
Magnesium-deficient	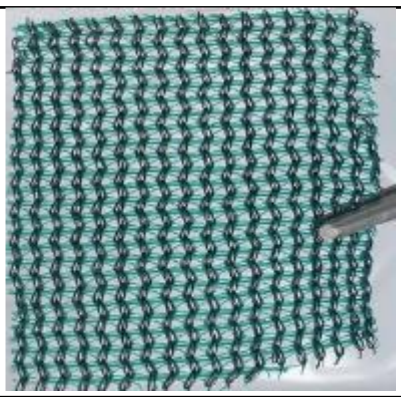	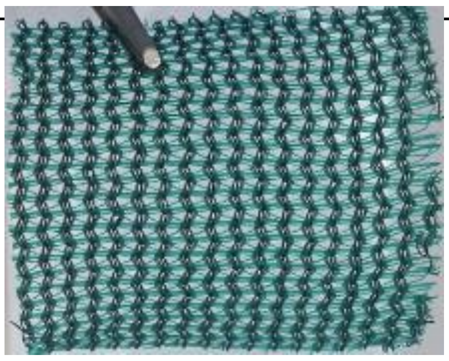
Sulphur-deficient	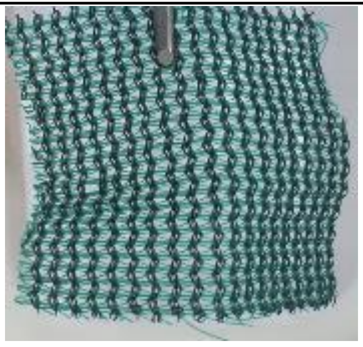	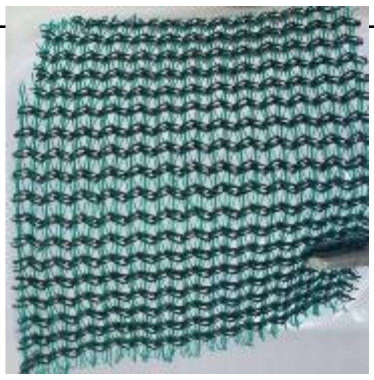
Phosphorous-deficient	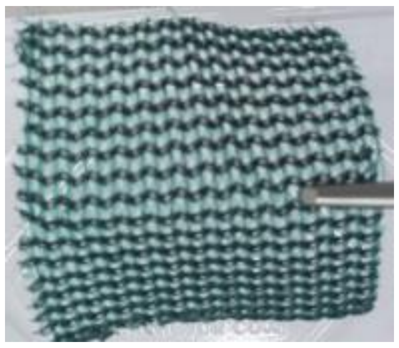	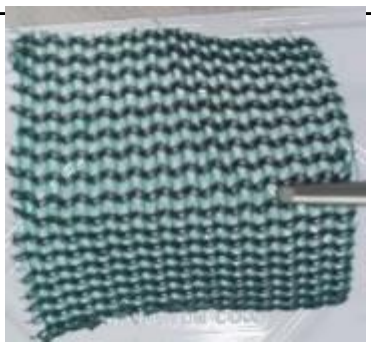

Day 32	Sample 1	Sample 2
Control	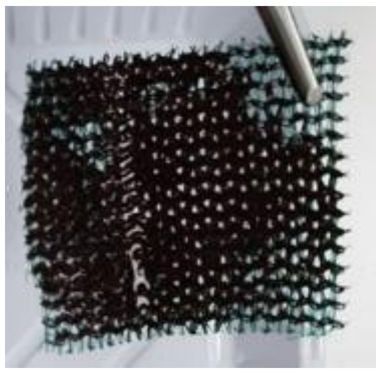	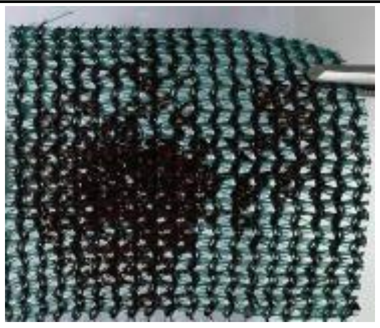
Nitrogen-deficient	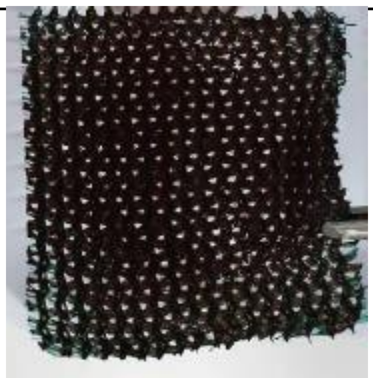	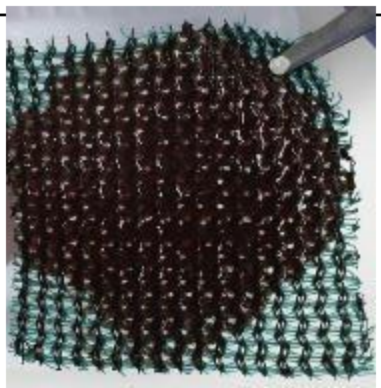
Calcium-deficient	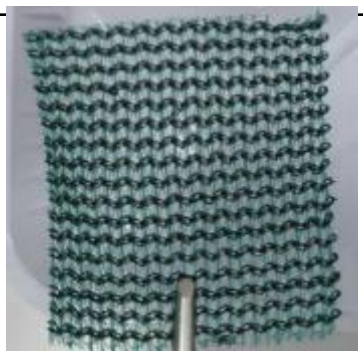	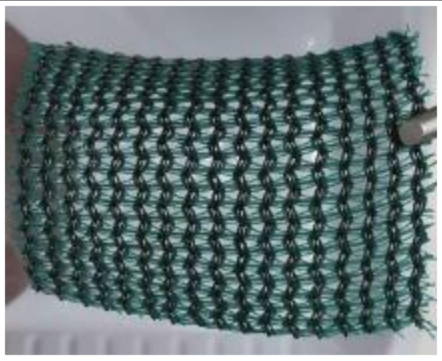
Magnesium-deficient	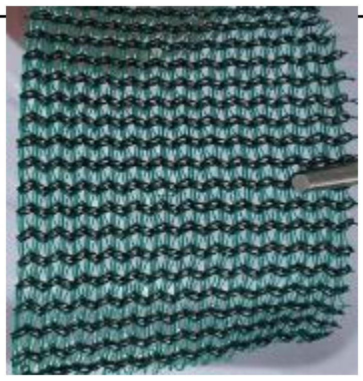	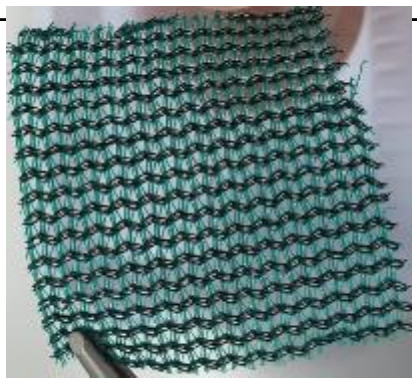
Sulphur-deficient	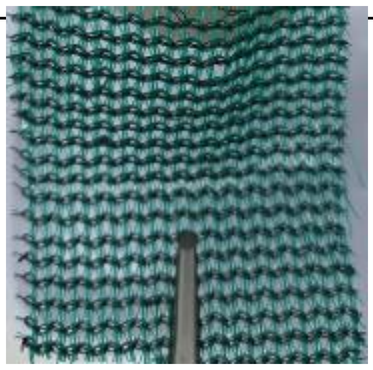	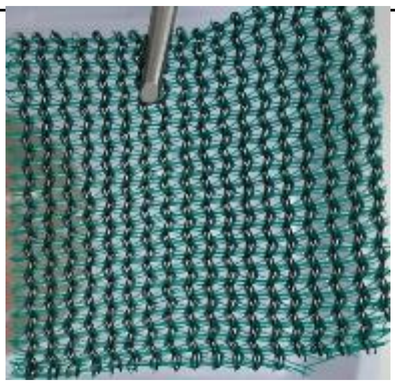
Phosphorous-deficient	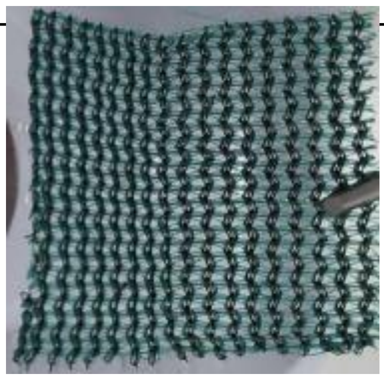	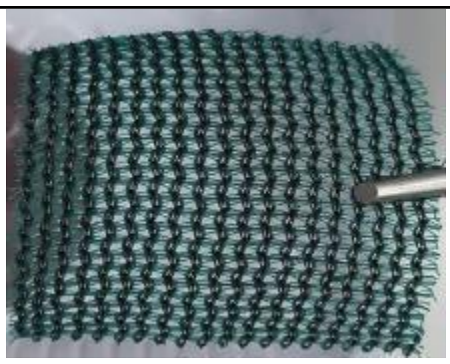
